# Synergistic effects of oncolytic reovirus and docetaxel chemotherapy in prostate cancer

**DOI:** 10.1186/1471-2407-11-221

**Published:** 2011-06-06

**Authors:** Lucy Heinemann, Guy R Simpson, Angela Boxall, Timothy Kottke, Kate L Relph, Richard Vile, Alan Melcher, Robin Prestwich, Kevin J Harrington, Richard Morgan, Hardev S Pandha

**Affiliations:** 1 Oncology, Postgraduate Medical School, University of Surrey, Guildford, GU2 7WG, UK; 2 Mayo Clinic, Rochester, USA; 3 Cancer Research UK Clinical Centre, St James's University Hospital, Beckett Street, Leeds, LS9 7TF, UK; 4 Targeted Therapy Team, Institute for Cancer Research, Chester Beatty Laboratories, 237 Fulham Road, London, SW3 6JB, UK

## Abstract

**Background:**

Reovirus type 3 Dearing (T3D) has demonstrated oncolytic activity *in vitro*, in *in vivo *murine models and in early clinical trials. However the true potential of oncolytic viruses may only be realized fully in combination with other modalities such as chemotherapy, targeted therapy and radiotherapy. In this study, we examine the oncolytic activity of reovirus T3D and chemotherapeutic agents against human prostate cancer cell lines, with particular focus on the highly metastatic cell line PC3 and the chemotherapeutic agent docetaxel. Docetaxel is the standard of care for metastatic prostate cancer and acts by disrupting the normal process of microtubule assembly and disassembly. Reoviruses have been shown to associate with microtubules and may require this association for efficient viral replication.

**Methods:**

The effects of reovirus and chemotherapy on *in vitro *cytotoxicity were investigated in PC3 and Du 145 cells and the interactions between agents were assessed by combination index analysis. An Annexin V/propidium iodide fluorescence-activated cell sorting-based assay was used to determine mode of cell death. The effects of reovirus and docetaxel administered as single agent or combination therapy were tested *in vivo *in a murine model. The effects of docetaxel and reovirus, alone and together, on microtubule stabilisation were investigated by Western blot analysis.

**Results:**

Variable degrees of synergistic cytotoxicity were observed in PC3 and Du 145 cells exposed to live reovirus and several chemotherapy agents. Combination of reovirus infection with docetaxel exposure led to increased late apoptotic/necrotic cell populations. Reovirus/docetaxel combined therapy led to reduced tumour growth and increased survival in a PC3 tumour bearing mouse model. Microtubule stabilization was enhanced in PC3 cells treated with reovirus/docetaxel combined therapy compared to other reovirus/chemotherapy combinations.

**Conclusions:**

The co-administration of a variety of chemotherapeutic agents with live reovirus was able to enhance cytotoxicity synergistically *in vitro*. The combination of docetaxel with reovirus also delayed tumour growth and improved survival *in vivo*. Enhanced microtubule stabilisation following this combination treatment may, in part, explain the mechanism of synergy. These results provide evidence to support the ongoing clinical trials using these agents.

## Background

Reoviruses (Respiratory Enteric Orphan viruses) are non-enveloped icosahedral viruses with a segmented double stranded RNA genome. Reoviruses are ubiquitous, non-pathogenic viruses that have innate oncolytic activity in a wide range of human and murine tumour cells. This property correlates with the transformed state of the cell [1,2] as transformation of immortalized cells which were not tumorigenic *in vivo *with oncogenes such as Ras, Sos, v-erb and c-myc rendered them susceptible to reovirus oncolysis [3-5]. In normal cells, activation of double-stranded RNA-activated protein kinase system (PKR) prevents significant viral replication; in malignant cells with an activated Ras pathway, up-regulated upstream or downstream components of the cell signaling pathway or up-regulated epidermal growth factor receptor signaling [3,4,6], this cellular antiviral response mechanism is perturbed and viral replication occurs leading to cytolysis of the host cell. In view of the high frequency of Ras dysregulation in different cancers [7], reovirus has potential as a broadly applicable anti-cancer therapeutic. A number of phase I clinical studies of intratumoral or systemic reovirus as a single agent have been completed, with evidence of significant antitumor activity [8,9]. However, in order to maximise the efficiency of tumour kill, combination therapy with other treatment modalities such as radiation or chemotherapy is likely.

Prostate cancer is one of the most common types of cancer in men, accounting for approximately 24% of new diagnoses and 13% of cancer deaths in the UK [10]. Surgery and radiotherapy may be curative, but significant numbers of patients relapse or present with locally advanced or metastatic disease and are treated with hormonal therapy. However, most subsequently progress and are treated with further hormonal therapy or chemotherapy.

Following several reports of significant activity in prostate cancer [11,12], docetaxel (taxotere) has become the standard of care first line chemotherapy agent worldwide. Docetaxel is a member of the taxane family and binds with high affinity to tubulin in microtubules, stabilising the microtubule and preventing depolymerisation [13-15]. Mitotic cell division is inhibited by the decrease in free tubulin, and the accumulation of microtubules within the cell leads to the initiation of apoptosis.

Reoviruses have been shown to associate with microtubules [16] via the core protein μ2 [17] and it has been proposed that efficient reovirus growth in some cell types may be dependent on μ2-mediated recruitment of viral factories to microtubules [18]. The stabilisation of microtubules by docetaxel could be expected to facilitate reovirus replication and enhance the therapeutic potential of the combination.

In this study we have examined the potential for synergistic or additive anticancer effects of combining reovirus with docetaxel in human prostate cell lines. We report this combination leads to enhanced cell death *in vitro *and reduced tumour growth *in vivo *providing evidence to support the ongoing clinical trials using these agents together.

## Methods

### Cell lines

The human prostate cancer-derived cell lines PC3, Du 145 and LNCaP were cultured in RPMI 1640 medium (Sigma-Aldrich, Gillingham, UK) at 37°C and 5% CO_2_. L929, a murine fibroblast-like line, was cultured in DMEM medium (Sigma-Aldrich) at 37°C and 5% CO_2_. All media were supplemented with 2 mM GlutaMAX-1 supplement (Invitrogen, Paisley, UK), 100 units/mL penicillin/streptomycin (Sigma-Aldrich) and either 10% (v/v) foetal calf serum (FCS; Invitrogen) for routine passage or 2% (v/v) FCS for experimental work.

### Reovirus stocks and chemotherapeutic agents

Reovirus type 3 Dearing strain Reolysin^® ^was obtained from Oncolytics Biotech. Inc. (Calgary, Canada). Virus stock titre and virus stability was measured by standard plaque assay of serially diluted samples on L929 cells. Six-well plates were seeded with 1 × 10^6 ^L929 cells per well and infected with dilutions of viral stocks. After 3 h incubation at 37°C, the virus solution was removed and the wells were overlaid with a 1:1 mixture of 2% SeaPlaque agarose (Cambrex Bio Science Rockland, Inc, ME) and 2 × MEM (Invitrogen) supplemented to a final concentration of 5% (v/v) FCS, 100 units/mL penicillin/streptomycin and 2 mM GlutaMAX-1. Wells were stained with 500 μL 0.03% neutral red (Sigma-Aldrich) in PBS 72 h post-infection and plaques were counted 3 to 4 h later.

Docetaxel (Sanofi-Aventis), paclitaxel (Bristol-Myers Squibb Company, N.Y.), vincristine sulphate (Tocris Bioscience) and cisplatin (cis diamminedichloroplatinum; Mayne Pharma Plc, UK) were all obtained from Royal Surrey County Hospital pharmacy. Doxorubicin hydrochloride was purchased from Sigma-Aldrich.

### *In vitro *survival assay

Cells were plated in 96-well plates at a density of 5 × 10^3 ^cells per well for PC3 and 7.5 × 10^3 ^cells per well for Du 145 and LNCaP. After 24 h, they were infected with known dilutions of reovirus, either alone or in combination with a chemotherapeutic agent. Control wells received an equivalent volume of assay medium. After 48 h incubation, cell viability was quantified using the CellTiter 96 AQueous One Solution Cell Proliferation Assay reagent 3-(4,5-dimethylthiazol-2-yl)-5-(3-carboxymethoxyphenyl)-2-(4-sulfophenyl)-2H-tetrazolium (MTS; Promega, Southampton, UK) according to manufacturer's instructions. Briefly, 20 μL of MTS reagent was added to each well and following incubation at 37°C for 1-4 h, absorbance was measured at 490 nm. Survival was calculated as a percent compared to untreated cells.

### *In vitro *synergy assay

The effect of the combination of reovirus and chemotherapy on cell proliferation was assessed by calculating combination-index (CI) values using CalcuSyn software (Biosoft, Ferguson, MO). Derived from the median-effect principle of Chou and Talalay [19], the CI provides a quantitative measure of the degree of interaction between two or more agents. A CI of 1 denotes an additive interaction, >1 antagonism and <1 synergy. Experiments were performed as described for the *in vitro *survival assay using 4, 2, 1, 0.5 and 0.25 times the calculated median effective dose (ED50) of each agent in a constant ratio checkerboard design.

### Inactivation of reovirus by UV-irradiation and heat

Reovirus was UV inactivated by exposing 50 μL aliquots of viral stock at 1.2 × 10^10 ^pfu/mL to 720 millijoules irradiation using a Stratalinker UV Crosslinker 2400 (Stratagene, LA Jolla, CA) to cross link viral RNA. Heat inactivation was performed by heating 200 μL aliquots of viral stock at 1 × 10^9 ^pfu/mL for 20 min at 60°C. *In vitro *survival and synergy assays with docetaxel were performed as described above using PC3 cells to compare the activity of inactivated virus to live virus.

### *In vivo *studies

All procedures were approved by United Kingdom Home Office (PL70/6521) and institutional boards. Mice were purchased from B&K Universal Ltd. The experiment was repeated three times, using six mice in each treatment group. Subcutaneous tumours were established in the flank of each mouse by injecting 1 × 10^7 ^PC3 cells in a volume of 100 μL Hanks Balanced Salt Solution (HBSS; Sigma-Aldrich). Animals were examined thrice weekly for tumour development. Three orthogonal tumour diameters (d1, d2, and d3) were measured using Vernier callipers and tumour volume was calculated from the formula V = π/6 d1·d2·d3. Animals were killed when tumour size exceeded 15 mm in any one dimension.

Once tumours were established and palpable, mice were randomly assigned to treatment groups and treated on days 0 and 3 with either reovirus or docetaxel alone or as a combined therapy. Reovirus (1 × 10^8 ^pfu in 100 μL volume) was administered using a single cutaneous puncture site. Once in a s.c. location, the 25-gauge needle was redirected along multiple tracks within the tumour to achieve maximal dispersal of the reovirus. Docetaxel (5 mg/kg) was administered intraperitoneally in a total volume of 100 μL. Vehicle control injections of 100 μL HBSS were administered in an identical manner to animals receiving single agent therapy and to control animals.

### FACS analysis of cell survival and apoptosis

Following overnight seeding, PC3 cells were treated with 20 nM docetaxel and/or reovirus MOI 1 for 48 h. Adherent and non-adherent cells were collected, washed in cold PBS, re-suspended at 1 × 10^6 ^in 500 μL PBS and then incubated for 15 min at room temperature in the dark in cold 1 × binding buffer containing Annexin V-FITC antibody, according to manufacturer's instructions (Merck Biosciences Ltd). The cells were pelleted and re-suspended in cold 1 × binding buffer. Cells were stained with 10 μL propidium iodide (PI) at 30 μg/mL and analysed on a Coulter Epics XL flow cytometer (Beckman Coulter) using EXPO32 ADC software (Beckman Coulter).

### Measurement of microtubule stability by Western blot analysis

PC3 cells were seeded overnight at 3 × 10^6 ^cells in 10 mL media in 10 cm Petri dishes and then treated with 5 nM docetaxel, reovirus at MOI 1, both, or neither for 48 and 72 h. Cells were washed twice in cold PBS and lysed in 500 μL cold RIPA buffer (Thermo Scientific) containing 5 μL of each of Halt protease inhibitor cocktail, phosphatase inhibitor cocktail and EDTA (Thermo Scientific). The samples were incubated on ice for 5 min prior to shearing of DNA by 3 to 4 passes through a 21ga needle. The samples were clarified by centrifugation and the supernatant was transferred to clean tubes and stored at -80°C prior to analysis by Western blot. Additional samples were collected from PC3 cells treated with paclitaxel (3.5 nM), cisplatin (1.25 μM), vincristine (40 nM) or doxorubicin (125 nM) alone or in combination with reovirus for 48 h.

Total protein (5 μg) was electrophoresed on 10% Bis-Tris gels (Invitrogen), transferred to polyvinylidene difluoride membranes, blocked, and exposed overnight to a mouse monoclonal acetylated α-tubulin primary antibody (6-11B-1, 1:30,000; Sigma-Aldrich) or mouse α-tubulin antibody (B-5-1-2, 1:50,000; Sigma-Aldrich) followed by incubation with a horseradish peroxidase labelled secondary antibody. Signal was developed using an Enhanced Chemiluminescence Plus Detection System (Amersham, UK).

### Effect of low-dose docetaxel on virus production

PC3 cells were infected with reovirus (MOI 1) alone or in combination with 5 nM docetaxel. Samples were collected at 24, 48, 72 and 96 h post infection and following three cycles of freeze-thaw, were tested for viral titre by plaque assay of 10-fold serial dilutions on L929 cells.

### Statistical analysis

Comparisons between groups were done using the 2-sided t-test and 2-way ANOVA. Statistical analysis was performed using GraphPad Prism 4 (GraphPad Software Inc.).

## Results

### Reovirus cytotoxicity in PC3, Du 145 and LNCaP cell lines

**Figure 1:**
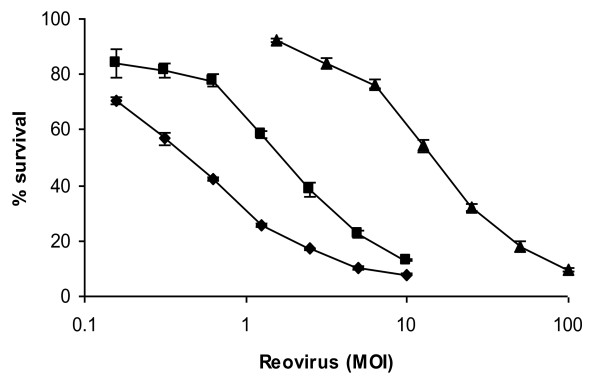
**Differing sensitivities of prostate lines to reovirus infection**. Prostate cell lines PC3 (diamonds), LNCaP (squares) and Du 145 (triangles) were infected with reovirus type 3 Dearing strain Reolysin at the indicated MOI for 48 h. Cell survival at this time was determined using MTS assay. Data are normalized to the uninfected control (MOI 0) for each cell line and are representative of three independent experiments.

The effect of reovirus infection over 48 h was assessed in three human prostate cancer cell lines. Cells were infected with doubling dilutions of reovirus from MOI 10 or 100. Differential sensitivity to reovirus across the cell lines was observed (Figure 1). PC3 cells were the most sensitive to reovirus induced cell death whereas Du 145 cells were the most resistant.

### Reovirus cytotoxicity is enhanced by combination with docetaxel

**Figure 2:**
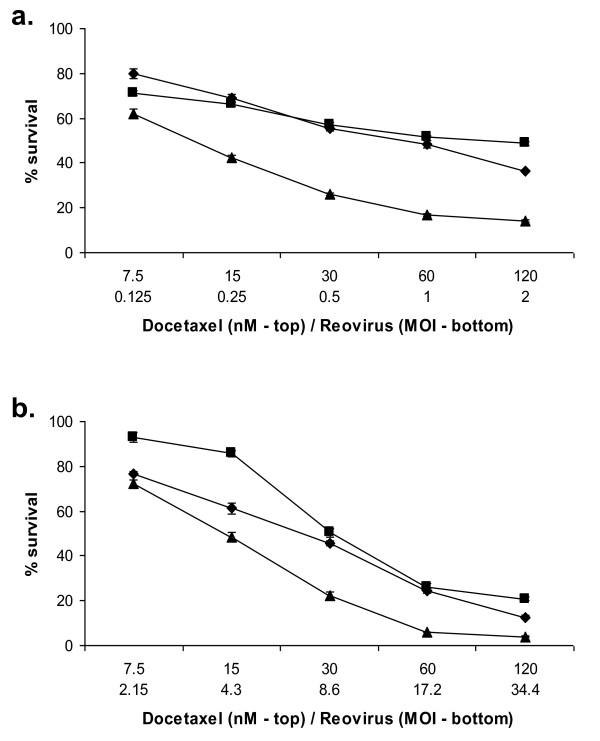
**Reovirus induced cell kill is enhanced by the addition of docetaxel**. PC3 (a) and Du 145 cells (b) were treated with reovirus alone (diamonds), docetaxel alone (squares) or a combination of the two (triangles) at the doses indicated for 48 h. Survival was measured at this time using MTS. In both cell lines tested, the combined treatment significantly reduced cell survival compared to either treatment alone (2-way ANOVA, p < 0.0001).

Using the data obtained above, the median effective dose (ED50) i.e. one at which half the test cells were killed at a given time point was calculated for reovirus. An ED50 for docetaxel was established in the same manner. PC3 and Du 145 cells, being the most and least susceptible cell lines respectively, were chosen for further study and treated with either reovirus or docetaxel alone, or in combination, at doses representing 4, 2, 1, 0.5 and 0.25 times the calculated ED50 in a constant ratio checkerboard design (Figure 2). An MTS survival assay was performed after 48 hours. The combination of agents enhanced tumour kill compared to each agent alone.

### Synergistic interaction between reovirus and chemotherapeutic agents in PC3 and Du 145 prostate cancer cell lines

From this data we calculated combination indices (CI) and used isobologram analysis to determine whether this enhancement of cell death could be considered synergistic. The CI provides a quantitative measure of the degree of interaction between two or more agents. A CI of 1 denotes an additive interaction, >1 antagonism and <1 synergy.

The combination of reovirus and docetaxel on PC3 cells was shown to be highly synergistic at the ED50 (Table 1), but becoming mildly antagonistic as the doses were increased. In contrast, on Du 145 cells, the combination proved to be additive at the ED50 (Table 2), becoming increasingly synergistic as the dose was increased.

**Table 1:** Interaction of reovirus and chemotherapy on PC3^a^

Chemotherapeutic	ED50	ED75	ED90
Docetaxel	0.41 ± 0.07	0.75 ± 0.04	1.58 ± 0.08
Paclitaxel	0.57 ± 0.01	0.40 ± 0.03	0.31 ± 0.04
Vincristine	0.46 ± 0.06	0.34 ± 0.05	0.44 ± 0.11
Cisplatin	0.89 ± 0.06	0.51 ± 0.07	0.33 ± 0.05
Doxorubicin	0.49 ± 0.03	0.22 ± 0.03	0.11 ± 0.03

^a^data is presented as combination index values ± SEM of three independent experiments at the effective dose indicated

**Table 2:** Interaction of reovirus and chemotherapy on Du 145^a^

Chemotherapeutic	ED50	ED75	ED90
Docetaxel	1.02 ± 0.05	0.83 ± 0.03	0.75 ± 0.07
Paclitaxel	0.89 ± 0.06	0.70 ± 0.08	0.58 ± 0.10
Vincristine	0.80 ± 0.01	0.57 ± 0.05	0.44 ± 0.08
Cisplatin	0.74 ± 0.13	0.61 ± 0.06	0.52 ± 0.06
Doxorubicin	0.82 ± 0.03	0.69 ± 0.03	0.59 ± 0.06

^a^data is presented as combination index values ± SEM of three independent experiments at the effective dose indicated

We then evaluated the effect of other chemotherapeutic agents on these two cell lines (Tables 1 and 2), looking at paclitaxel (another taxane), vincristine (a microtubule inhibitor), cisplatin (a pseudo-alkylating agent) and doxorubicin (a DNA intercalating agent). We observed synergistic cell kill with all agents, however CI values were slightly lower in PC3 cells than in Du 145.

### Live virus is required for efficient cell killing

**Figure 3:**
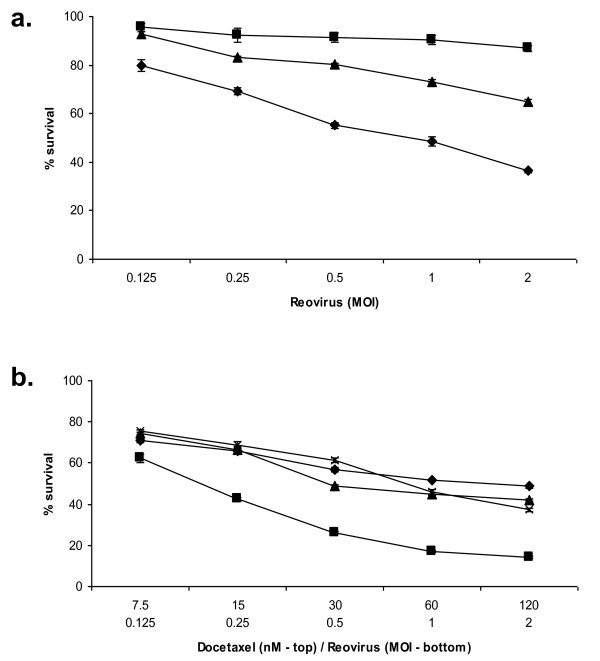
**Live virus is required for efficient cell kill and enhanced cell kill with docetaxel**. PC3 cells were treated with live (diamonds), heat-inactivated (squares), or UV-inactivated (triangles) virus for 48 h at the MOI indicated at which point survival was measured by MTS assay (a). PC3 cells were also treated with docetaxel alone (diamonds), or docetaxel with live reovirus (squares), heat-inactivated reovirus (triangles) or UV-inactivated virus (crosses) for 48 h at the concentration and MOI indicated. Survival was measured at this time by MTS assay. The combination of live reovirus with docetaxel enhanced cell kill compared with docetaxel alone (P < 0.0001, two-way ANOVA), however this enhancement of cell kill was not apparent when heat inactivated or UV-inactivated reovirus was used (b). Data for both are normalized to uninfected controls (MOI 0) and are representative of three independent experiments.

Aliquots of reovirus were exposed to heat or UV irradiation to inactivate the virus and then used alongside live virus in an MTS survival assay using PC3 cells. Live virus resulted in the most efficient cell kill, ranging from 80 to 36% survival at the MOIs used. This effect was markedly reduced when the same MOI of UV-inactivated virus was used, and almost completely negated by heat inactivated virus (Figure 3a).

Live, heat inactivated and UV-inactivated reovirus was combined with docetaxel at a fixed ratio of 60 nM docetaxel to MOI 1 reovirus over five two-fold dilutions for 48 hours. The combination of live reovirus with docetaxel enhanced cell kill compared to docetaxel alone (2-way ANOVA p < 0.0001). This enhancement of cell kill was not apparent when heat inactivated or UV-inactivated reovirus was used (Figure 3b)

### Combined reovirus and docetaxel treatment delays tumour growth in a PC3 xenograft murine model

In light of the observed *in vitro *synergy, the effects of combined reovirus and docetaxel on PC3 cells were evaluated *in vivo *in a nude mouse model. PC3 cells (1 × 10^7^) were implanted subcutaneously in the flanks of nude mice and treatment was initiated when tumours reached an average diameter of 3 mm. Mice were treated with intratumoral reovirus, intraperitoneal docetaxel or both on day 0 and day 3. Control mice received the same tumour inoculum and were treated with an equivalent volume of HBSS i.t. and i.p. administered in an identical manner. There were no obvious toxic effects of single agent or combination treatments in all mice treated, and experiments were concluded as a result of tumour growth reaching 15 mm in any one dimension.

**Figure 4:**
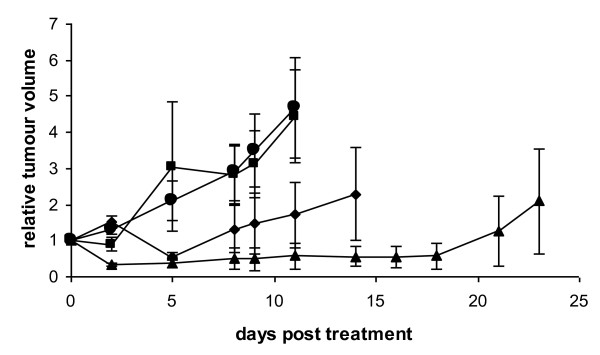
**Reduced tumour growth and increased survival following reovirus/docetaxel combination therapy**. Nude mice bearing subcutaneous PC3 tumours were treated on days 0 and 3 with either 1 × 10^8 ^pfu reovirus alone i.t. (diamonds), 5 mg/kg docetaxel alone i.p. (squares), or reovirus and docetaxel in combination (triangles). Control treated mice (circles) and single therapy treated mice received equivalent HBSS vehicle control injections. Tumours were measured on the days indicated and tumour volume expressed as tumour volume relative to volume at commencement of treatment. Docetaxel alone had no effect on tumour volume compared to control treatment (p > 0.05). Reovirus alone had a moderate effect (p < 0.05 day 9 and p < 0.001 day 11), but the greatest effect was when reovirus therapy was combined with docetaxel (p < 0.05 day 8, p < 0.01 day 9 and p < 0.001 day 11). Mice were euthanized when tumours exceeded 15 mm in any one dimension. Data shown are representative of three independent experiments.

The combination of docetaxel and reovirus resulted in the most effective response in terms of tumour growth retardation (p < 0.001, day 11, Figure 4), although reovirus monotherapy also resulted in delayed tumour growth (p < 0.001, day 11). Docetaxel monotherapy however had no effect on tumour growth at this dose (p > 0.05).

### Enhanced apoptotic cell death with docetaxel and reovirus combination

**Figure 5:**
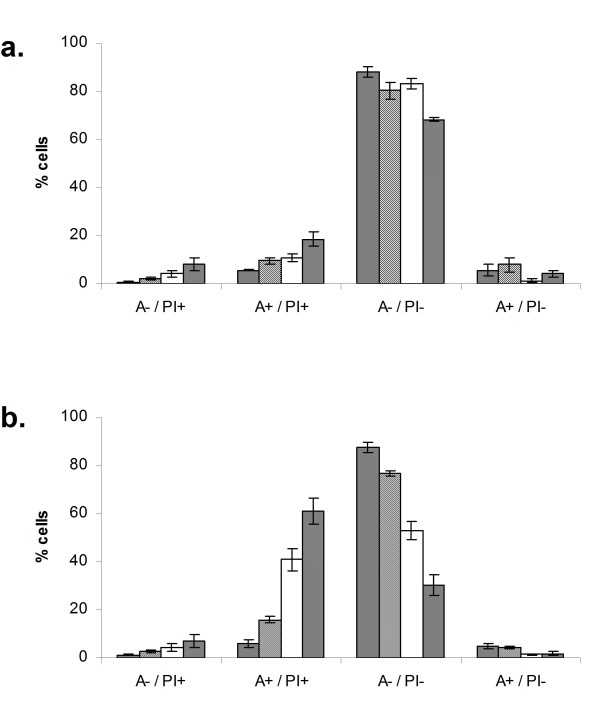
**Enhanced apoptotic cell death of PC3 cells treated with reovirus and docetaxel in combination**. PC3 cells were treated with reovirus MOI 1 (open bars), 20 nM docetaxel (wide diagonal stripes) or both agents together (narrow diagonal stripes) for 24 h (a) or 48 h (b) before staining with Annexin V (A) and propidium iodide (PI) and analysing using flow cytometry. Untreated cells (dark grey bars were included as a negative control). Addition of each agent alone resulted in an increased late apoptotic/necrotic population (A+/PI+) and concomitant reduction of intact cells (A-/PI-) compared to untreated cells. There was a dramatic increase in this effect at 48 h when the two agents were combined.

We wished to investigate further the nature of the synergy of cell kill with reovirus and docetaxel treatment. The mode of cell death of PC3 cells treated with reovirus MOI 1, docetaxel 20 nM or both agents together was assessed at 24 and 48 h by annexin/PI staining. At 24 hours, there was a small increase in late apoptotic/necrotic population (A+PI+) in all groups, but slightly more so in the combination. By 48 h, this effect had increased considerably with the majority of cells in the combination group A+PI+ and concomitant reduction of intact cells (A-PI-). The effect of reovirus alone also caused a degree of apoptotic death in this cell line (Figure 5a and 5B).

### Increased acetylation of microtubules following exposure to reovirus and docetaxel

**Figure 6:**
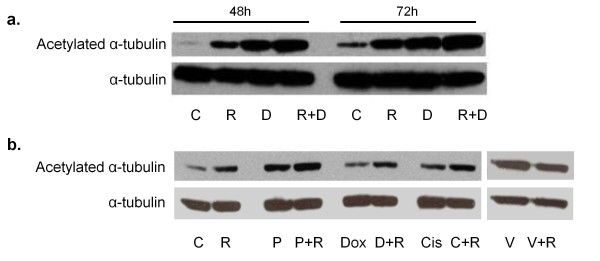
**Enhanced stabilisation of microtubules**. PC3 cells were treated with docetaxel (D) or reovirus (R), alone or in combination (R+D) for 48 or 72 h (a) and levels of α-tubulin and acetylated α-tubulin were assessed by Western blot. Both treatments alone led to an increase in acetylated α-tubulin compared to untreated cells (C). When applied in combination, there was an even greater increase. Levels of acetylated α-tubulin increased for each treatment in a time dependent manner. Protein samples were additionally collected at 48 h post treatment from PC3 cells treated with paclitaxel (P), doxorubicin (Dox), cisplatin (Cis) and vincristine (V) and assessed in the same way (b). Treatment with paclitaxel and vincristine led to an increase in acetylated α-tubulin above untreated cells, whereas treatment with doxorubicin and cisplatin did not. Only treatment with paclitaxel in combination with reovirus produced levels of acetylated α-tubulin greater than that of reovirus infection alone.

Docetaxel is known to enhance microtubule stability, ultimately leading to apoptotic cell death. The acetylation of α-tubulin may be used as a marker of microtubule stability, with the amount of acetylated α-tubulin being proportional to the stability of the microtubule. We wished to determine if the combination of reovirus and docetaxel had any enhancement of effect on acetylated α-tubulin expression compared to each agent alone. Protein samples from PC3 cells were collected at 48 and 72 h after treatment with docetaxel or reovirus alone or in combination. Treatment with docetaxel alone led to a substantial increase in acetylated α-tubulin compared to untreated cells at both time points. An increase in acetylated α-tubulin was also observed in samples from cells infected with reovirus alone. Docetaxel in combination with reovirus led to the greatest increase, suggesting an additive or possibly synergistic effect (Figure 6a). We then looked at the effect of other chemotherapeutic agents on acetylated α-tubulin induction in PC3 cells. Background levels of acetylated α-tubulin were detected following exposure to cisplatin and doxorubicin. Exposure to paclitaxel and vincristine (both know to stabilise microtubules) resulted in an above background level of acetylated α-tubulin. When combined with reovirus however, only cells exposed to the paclitaxel/reovirus combination exhibited levels of acetylated α-tubulin greater than following exposure to reovirus alone (Figure 6b).

### Increased viral titre at early time points from cells exposed to reovirus and docetaxel compared to reovirus alone

**Figure 7:**
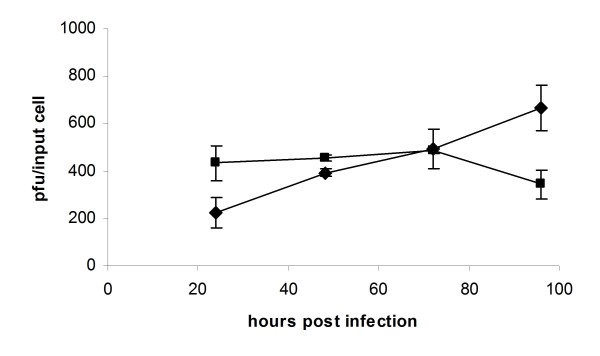
**Effect of low-dose docetaxel on viral production in PC3 cells**. PC3 cells were infected with reovirus at MOI 1 alone (diamonds) or in combination with 5 nM docetaxel (squares). Triplicate samples were collected at the time points indicated and virus amount determined by plaque assay.

Samples were collected at 24, 48, 72 and 96 h post infection from PC3 cells infected with reovirus in association with low dose docetaxel and virus titre was determined by plaque assay. A higher titre was recovered at earlier time points from cells treated with docetaxel and reovirus compared to cells infected with reovirus alone. By 72 h post infection however, there was no difference in titre and at 92 h post infection, while the titre in reovirus only samples continued to increase, it was decreasing in samples collected from cells treated with both agents (Figure 7).

## Discussion

The modest improved survival with docetaxel chemotherapy in recent studies has been a significant step forward in the treatment of hormone refractory metastatic prostate cancer (HRPC) but the overall poor prognosis and morbidity justifies the continued development of new treatment approaches [11,12]. Although a number of oncolytic viruses have shown significant anti-tumour effects in phase I clinical studies [8,9,20,21], their potential as main stream anti-cancer therapeutics will most likely be realised as combination therapy with other cancer treatment modalities. Theoretically, combination therapy could potentially allow reovirus to target drug or radiotherapy resistant subpopulations of tumour cells, or chemotherapy could be utilised to improve the biodistribution of oncolytic viruses.

In this study, we have demonstrated evidence of synergistic anti-cancer activity of oncolytic wild type reovirus with docetaxel in human prostate cancer *in vitro *and *in vivo*. *In vitro *synergy was further observed with other chemotherapeutic agents. The potential value of reovirus in combination with docetaxel was tested in a murine flank model of human prostate cancer. We did not observe any toxic effects in the treatment groups and while docetaxel alone had little effect on tumour progression at the dose used, reovirus alone had a modest effect. In combination however, there was significant inhibition of tumour progression.

Docetaxel and other members of the taxane family of chemotherapeutic drugs have been shown to stabilise microtubules in mitotic cells. Microtubules are essential for the separation of the duplicated chromatids to opposing poles prior to mitotic cell division. Stabilisation of microtubules impairs normal changes in microtubule structure, leading to a block in mitosis and promotion of apoptosis [22,23]. The reoviral μ2 protein is an approximately 83 kDa protein encoded by the M1 genome segment. It forms a structurally minor component of the reovirus core and binds ssRNA and dsRNA [24-27]. It is also capable of binding to cellular microtubules [17,28,29]. It has been proposed that for some cell types, μ2-mediated recruitment of viral factories to microtubules might be necessary for efficient reovirus growth [18]. We proposed that the presence of another agent also stabilising microtubules, could enhance these effects. We found that PC3 cells treated with reovirus in combination with docetaxel exhibited a considerable increase in microtubule acetylation compared to untreated or single agent treated cells. The same effect was observed with paclitaxel but not with doxorubicin or cisplatin which do not alter microtubule stability. Interestingly we found no enhancement of microtubule stability with vincristine, but this can be explained by the known mechanisms of action of the vinca alkaloids on tumour cells. The vinca alkaloids, such as vinblastine, vincristine, and vinorelbine, bind to the end of growing microtubules, blocking the addition of more tubulin dimers. The tubule cannot grow, but it can still disassemble, so the microtubules ultimately break down. The inhibition of tubular growth by vincristine would therefore not allow microtubule stabilisation, reovirus adherence and reoviral replication. However, our *in vitro *studies showed that the combination of vincristine and reovirus still resulted in synergistic cell kill suggesting some alternative mechanism. These findings provide further evidence of multiple mechanisms by which reovirus may combine with other anti-cancer treatments to enhance its antitumour effects. This broadens the potential clinical utility of reovirus very greatly. Although the data from PC3 cells was most compelling in terms of synergy by CI, we did see evidence of at least additive effects in DU145 with docetaxel and synergy with paclitaxel rather than antagonism. It would be important to evaluate the *in vivo *effects of combination with vincristine and paclitaxel to confirm the *in vitro *findings in future work. There would also be an opportunity to assess post-treatment tissue for reovirus replication and microtubule protein expression to compare our *in vitro *findings

In this study, we found an increase in viral titre at early time points in cells treated with the combined therapy. This has been noted previously for various non-small cell lung cancer cell lines treated with paclitaxel in combination with reovirus [30]. At later time points, as the cells treated with docetaxel and reovirus became apoptotic and necrotic, the amount of virus recovered fell to levels less than from cells infected with reovirus alone.

Other chemotherapy/oncolytic virus combinations have also shown considerable promise. Recently, G47delta, an engineered oncolytic herpes simplex virus-1 was shown to have synergistic antitumour effects *in vitro *and *in vivo *in combination with taxanes through the enhancement of apoptosis [31]. The adenovirus Onyx-015 enhanced clinical efficacy when used as intratumoral injection combined with systemic cisplatin and 5-fluorouracil (5FU) compared to chemotherapy alone [32]. In preclinical models, synergy has been demonstrated with the combination of E1A-expressing adenoviral E3B mutants with cisplatin and paclitaxel [33], rat parvovirus H-1PV with gemcitabine [34] and oncolytic herpesviruses such as G207, HSV-1716, and NV1066 with various chemotherapeutic agents [35-37]. The mechanism underlying the observed synergy is incompletely understood and as in our study, not necessarily due to increased viral replication [38-40].

Recently completed phase I studies by our group and others using Reovirus type 3 (Dearing) have confirmed its potential as an anticancer agent as well as its safety and tolerability in humans [8,9]. This has led to combination studies of systemic reovirus with a number of chemotherapeutic agents and radiotherapy as phase I studies (REO-). These include attempts to enhance cytotoxicity with gemcitabine (REO 09), docetaxel (REO10) and carboplatin/paclitaxel (REO11, REO15 and REO16) in a number of indications. The REO10 study showed the reovirus/docetaxel combination was safe and a maximum tolerated dose was not reached. Antitumor activity was seen with one complete response and three partial responses. A disease control rate (combined complete response, partial response, and stable disease) of 88% was observed. Immunohistochemical analysis of reovirus protein expression was observed in post-treatment tumor biopsies from three patients [41].

## Conclusions

Reovirus has a potentially broad application as an anti-cancer therapeutic and oncolytic activity has been demonstrated *in vitro*, in *in vivo *murine models and in early clinical trials. Administration in combination with other modalities such as chemotherapy, targeted therapy and radiotherapy may be necessary in order to realize the full potential of reovirus, and indeed other oncolytic viruses.

We have demonstrated here and elsewhere [42] that co-delivery of reovirus with chemotherapeutic agents with a variety of modes of actions is able to enhance *in vitro *cytotoxicity in a synergistic manner. Our focus here was the interaction of reovirus with docetaxel, the standard of care first line chemotherapy agent for prostate cancer, on the highly metastatic human prostate cell line PC3. We showed that the combination therapy was synergistic *in vitro*, it was capable of slowing tumour growth and prolonging survival in a PC3 tumour mouse model. Microtubule stabilisation was enhanced in PC3 cells following treatment with combined reovirus/docetaxel combined therapy which may, in part, explain the mechanism of synergy. This data supports the positive clinical observations from the recent REO10 docetaxel/reovirus combination study.

## Competing interests

The authors declare that they have no competing interests.

## Authors' contributions

All authors have read and approved the final manuscript. The authors made the following contributions to this work: LH conducted lab based experimental work and wrote the manuscript, GS carried out the animal work, AB carried out the Western blots, TK also carried out animal work, AM helped to write the manuscript, KR edited the paper, RV was involved in experimental design, AM was involved in experimental design and critique, RP, KH, RM and HP were involved in experimental design and critique. All authors read and approved the final manuscript.

## Pre-publication history

The pre-publication history for this paper can be accessed here:

http://www.biomedcentral.com/1471-2407/11/221/prepub
